# Purification and Characterisation of Two Novel Pigment Proteins from the Carapace of Red Swamp Crayfish (*Procambarus clarkii*)

**DOI:** 10.3390/foods11010035

**Published:** 2021-12-23

**Authors:** Hao Chen, Hongwu Ji, Chuang Pan, Di Zhang, Weiming Su, Shucheng Liu, Yijia Deng, Xiaodan Huang

**Affiliations:** 1 Guangdong Provincial Key Laboratory of Aquatic Product Processing and Safety, College of Food Science and Technology, Guangdong Ocean University, Zhanjiang 524088, China; spgc13@163.com (H.C.); zjs578180838@sina.com (D.Z.); hdsuwm@163.com (W.S.); Lsc771017@163.com (S.L.); ikea7713@163.com (Y.D.); 19875907745@163.com (X.H.); 2 Hunan Provincial Key Laboratory of Soybean Products Processing and Safety Control, College of Food and Chemical Engineering, Shaoyang University, Shaoyang 422000, China; 3 Guangdong Provincial Engineering Technology Research Center of Seafood, College of Food Science and Technology, Guangdong Ocean University, Zhanjiang 524088, China; 4 Guangdong Province Engineering Laboratory for Marine Biological Products, College of Food Science and Technology, Guangdong Ocean University, Zhanjiang 524088, China; 5 Key Laboratory of Advanced Processing of Aquatic Product of Guangdong Higher Education Institution, College of Food Science and Technology, Guangdong Ocean University, Zhanjiang 524088, China; 6 Collaborative Innovation Center of Seafood Deep Processing, Dalian Polytechnic University, Dalian 116034, China; silverpfoxc@hotmail.com; 7 South China Sea Fisheries Research Institute, Chinese Academy of Fishery Sciences, Guangzhou 510300, China

**Keywords:** pigment protein, cDNA, red shift properties, red swamp crayfish

## Abstract

Pigment proteins play a vital role in the red colour change of the red swamp crayfish (*Procambarus clarkii*) shell after cooking. In this study, two red-change-related pigment proteins with molecular weights of approximately 170 and 43 kDa—denoted as F1 and F2, respectively—were purified by ammonium sulphate salting-out and size exclusion chromatography. F1 and F2 entirely comprised homomultimeric protein complexes composed of 21 kDa subunits. LC-MS/MS analysis showed that the 21 kDa protein subunit belonged to the crustacyanin family, named *P*. *clarkii* crustacyanin A2 (PcCRA2). The full-length cDNA of PcCRA2 was cloned, which encoded 190 amino acid residues and was highly homologous (91.58%) with *Cherax quadricarinatus* crustacyanin A. The predicted 3D structure showed that PcCRA2 had a β-barrel structure for pigment encapsulation. The colour change of F1 was first detected at 40 °C, and the red change occurred upon heating above 60 °C. Additionally, with increasing temperature, its β-sheet content increased, and its α-helix content reduced. Correlation analysis showed that the redness value of F1 was significantly related to the heating temperature and the β-sheet content.

## 1. Introduction

During the processing and consumption processes, colour is an important indicator of the quality of a crustacean product, and it directly influences its acceptability and desirability to consumers [1,2]. In China, the red cooked Chinese mitten crab (*Eriocher sinesis*) draws consumers’ attention and acquires higher market prices than dark orange or yellow crabs [3]. A survey on colour preferences for prawns revealed that brightly orange cooked shrimps were preferred by 54.3% of respondents, whereas the lighter ones were rejected by 88.6% [1]. Additionally, cooked black tiger prawns are classified by Australians into 12-grade scores based on their red surface colour. Generally, prawns with higher grade scores are sold for AUD 2–4/kg higher than those with lower grade scores [4]. Thus, improving the red surface colour is beneficial to crustacean products’ commercial value.

Previous studies have demonstrated that the dissociation of astaxanthin (3,3′-dihydroxy-β,β′- carotene-4,4′-dione, AXT) from its pigment proteins in the carapace is closely related to the red surface colour change in crustaceans during cooking [5,6,7,8,9,10]. Natural pigment protein complexes have various colours, ranging from red-orange to blue-purple due to the interaction of AXT and different types of apoprotein [8,11]. The deposition of these pigment proteins can lead to diverse carapace colours and patterns [12]. It has traditionally been considered that the degree of crustacean surface red colour depends mainly on the total pigment-protein content; however, some studies have indicated that there is no correlation between the two [12,13]. A possible explanation is that pigment proteins have remarkable differences in molecular weight, thermal denaturation, temperature sensitivity, and other properties [10,14]. Ando et al. successfully improved the red colour of cooked kuruma prawns (*Marsupenaeus japonicus*) by altering the processing conditions based on the properties of pigment proteins [15].

Pigment proteins were first extracted from the carapace of the European lobster (*Homarus gammarus*) and were classified as crustacyanin A (CR-A) and crustacyanin C (CR-C), depending on their amino acid composition and peptide mapping [16,17]. Subsequent research found that one or more kinds of crustacyanin are present in most crustaceans, including shrimps, lobsters, and crabs [18]. Interestingly, in the shell of the Pacific white shrimp (*Penaeus vannamei*), not only crustacyanins A and C but also haemocyanin could bind with pigments [7,18]. However, a pigment-binding haemocyanin sample (1 mg·mL^−1^) was heated at 100 °C and had a redness CIELAB colour space value (a*) of only 0.21 [7]. Similarly, the a* value of pigment protein derived from *M*. *japonicus* reached 0.76 [19].

The red swamp crayfish (*Procambarus clarkii*) has become the most popular commercially harvested shrimp species in China due to its attractive bright red colour and delicious flavour. In 2020, the total harvest of *P. clarkii* in China reached 2.4 million tonnes, and its annual industrial output exceeded CNY 300 billion [20] (pp. 24–25). The crayfish from different farms or growing stages have various colours, ranging from brownish-green to dark-red, indicating the presence of diverse pigment proteins. Moreover, there is no significant difference in astaxanthin content among different coloured crayfish carapaces, but the red colour of brownish-green crayfish is substantially greater than that of dark-red crayfish (data is not shown). In our previous study, two pigment-binding proteins with molecular weights of 24 and 73 kDa were found in dark-red crayfish carapaces and identified as haemocyanins [21]. When the concentration of haemocyanins increased to 5 mg·mL^−1^, the a* value of 24 kDa protein increased from 2.10 to 3.26, whereas in the case of 73 kDa protein, this value increased from 10.28 to 16.57 in a boiling water bath. These results indicated that the pigment-binding haemocyanins were not red enough to achieve the red colour change in the brownish-green crayfish shell after cooking. Until now, no studies have been reported on crustacyanin derived from *P. clarkii* and its thermal denaturation. Furthermore, understanding red-change-related pigment proteins will help target measures to improve the colour and value of crustacean products during processing. Thus, the objective of this study was to purify and characterise the potential red-change-related pigment protein from brownish green *P. clarkii*.

## 2. Materials and Methods

### 2.1. Material and Chemicals

Red swamp crayfish (*P. clarkii*, *n* = 475, 11 ± 1 cm long and 32 ± 4 g in weight) were purchased from Qianjiang City, Hubei Province, China. A plastic foam box was used to transport the crayfish on ice to the Guangdong Provincial Key Laboratory of Aquatic Product Processing and Safety. The carapaces of the cephalothoraxes were collected, washed in ice water, and placed overnight in a cold room (4 °C) to remove surface moisture. Following this, they were intermittently ground into a fine powder (6.35 mm mesh) with an LD-T400 food blender (Dingshuai Electric Appliance Inc., Shanghai, China) to avoid degrading the heat-labile components.

Boric acid, Tris, sodium dihydrogen phosphate, disodium hydrogen phosphate, ethylenediaminetetraacetic acid (EDTA), sodium hydroxide, ammonium sulphate, and sodium chloride were of analytical grade and purchased from Xilong Scientific Co., Ltd., Shantou, China.

### 2.2. Crude Protein Preparation

Crude pigment proteins from *P. clarkii* carapaces were extracted in accordance with the method described by Zagalsky et al., with some modifications [22]. Briefly, carapace powder (25 g) was dissolved in 0.3 M boric acid with pH adjusted to 6.8 with solid Tris (1000 mL) and continuously mixed for 12 h by an MS300 magnetic stirrer (Bante Instrument Inc., Shanghai, China). The mixture was then centrifuged at 16,000× *g* for 20 min to remove the supernatant. The residue was extracted at 4 °C with a 10% (*w/v*) EDTA solution with pH adjusted to 7.0 with sodium hydroxide (250 mL) for 4 h and then centrifuged at 16,000× *g* at 4 °C for 20 min. The supernatant was collected, and the residue was re-extracted until the supernatant became colourless. The multiple supernatants (crude pigment proteins) were mixed and used for subsequent purification.

### 2.3. Targeted Protein Purification

The crude pigment proteins (1.5 mg·mL^−1^) were divided into 8 aliquots of 10 mL each, saturated with powdered ammonium sulphate ([NH_4_]_2_SO_4_) (10%, 20%, 30%, 40%, 50%, 60%, 70%, and 80% *w/v*) and then stirred overnight at 4 °C. After centrifugation at 16,000× *g* at 4 °C for 20 min, each resulting supernatant was collected separately, and the resulting precipitate was dissolved in sodium phosphate buffer (20 mM, pH 7.0, 10 mL). The total protein content of each supernatant and precipitate was measured using the Bradford protein assay kit (Beyotime Biotechnology, Shanghai, China), and bovine serum albumin was used as a reference. The colour change of each supernatant and precipitate was measured after heating at 100 °C for 10 min.

The precipitate obtained by the [NH_4_]_2_SO_4_ saturation of 20–60% was dissolved in sodium phosphate buffer (pH 7.0, 20 mM). The resulting primary pigment proteins were further purified by size exclusion chromatography (SEC) using an ÄKTA purifier high-performance liquid chromatography system (Superdex 200 column (16 × 100 mm; GE Healthcare Bio-Sciences AB, Staffanstorp, Sweden)). In short, the column was equilibrated and eluted with NaCl (0.15 M) and sodium phosphate buffer (20 mM) to adjust the pH to 7.0. The flow rate was 1.0 mL·min^−1^. Fractions were collected according to a real-time peak value at 280 nm, the concentration was adjusted to 1 mg·mL^−1^, and the colour change was observed after heating at 100 °C for 10 min. The purity of the eluted fractions was determined by sodium dodecyl sulphate–polyacrylamide gel electrophoresis (SDS-PAGE).

### 2.4. Molecular Weight Determination

The molecular weight of native purified pigment proteins was assessed using size exclusion chromatography (SEC). The sample (1 mg·mL^−1^, 20 μL) was loaded onto a TSK-gel G3000SWxl column (7.8 × 300 mm, Tosoh, Tokyo, Japan), eluted with sodium phosphate buffer (20 mM, pH 7.0) at a flow rate of 0.7 mL·min^−1^, and monitored at UV 280 nm. In addition, protein standard mix (including thyroglobulin (670 kDa), γ-globulins from bovine blood (150 kDa), albumin chicken egg grade VI (44.3 kDa), ribonuclease A (13.7 kDa), and p-aminobenzoic acid (PABA), Sigma-Aldrich, St. Louis, MO, USA) were used to calibrate the column.

### 2.5. Colour Measurement

The concentration of each obtained primary pigment proteins was adjusted to 5 mg·mL^−1^. For each protein solution, 0.5 mL aliquot was added into each of nine test tubes, which were then capped and heated in a water bath for 10 min at 25, 30, 40, 50, 60, 70, 80, 90, and 100 °C. The test tubes were then placed in iced water to cool to 25 °C.

Colour variations were assessed by a chroma meter (CR-20, Konica Minolta, Tokyo, Japan), and the lightness parameter (L*), redness (a*, a* > 0 red, a* < 0 green), and yellowness (b*, b* > 0 yellow, b* < 0 blue) were recorded. The total colour difference (dE) was calculated according to Equation (1):

(1) $$\mathrm{dE}={\left[{\left(L^{*}-L_{0}^{*}\right)}^{2}+{\left(a^{*}-a_{0}^{*}\right)}^{2}+{\left(b^{*}-b_{0}^{*}\right)}^{2}\right]}^{{}^{1/2}}$$

### 2.6. SDS-PAGE Analysis

The above thermally treated primary pigment-protein samples were centrifuged at 10,000× *g* for 10 min at 4 °C. The supernatants and precipitates were collected and analysed using SDS-PAGE in a 5% stacking gel and a 12% separating gel. Before electrophoresis, the precipitate was suspended in 0.5 mL of deionised water and homogenised by a vortex mixer at 2800 rpm (XW-80 A, Huxi Analysis Instrument Factory Inc., Shanghai, China). The gels were stained using a Coomassie blue staining solution (Beyotime Biotechnology, Shanghai, China) and scanned using a gel imaging system (GelDoc XR+, Bio-Rad, Hercules, CA, USA).

### 2.7. LC-MS/MS Analysis

The target protein bands in the stained SDS-PAGE gels were manually excised and de-stained by acetonitrile (50%), then treated with dithiothreitol (10 mM) and iodoacetamide (55 mM), and finally digested overnight at 37 °C with sequencing-grade trypsin (10 ng·nL^−1^; Promega, Madison, WI, USA). The digested solutions were subjected to peptide sequencing analysis by LC-MS/MS with a Q Exactive Orbitrap HF mass spectrometer with a nanoelectrospray ionization source (ThermoFisher Scientific, Waltham, MA, USA) and an LC-20AD nano high-performance liquid chromatography (HPLC) system equipped with a MonoCap C18 trap column (0.2 × 50 mm, 5 μm; Shimadzu, Tokyo, Japan).

The HPLC mobile phase comprised solutions A (5% acetonitrile containing 0.1% formic acid) and B (95% acetonitrile containing 0.1% formic acid). Samples were loaded by mobile phase A at a flow rate of 8 μL·min^−1^ for 4 min. The gradient elution program was as follows: 0 min, 2% B; 40 min, 35% B; 45 min, 80% B; 49 min, 80% B; 50 min, 2% B; and 60 min, 2% B at a flow rate of 300 nL·min^−1^.

The resulting nanoflow liquid chromatography eluate was directly subjected to nanoelectrospray ionization, followed by mass spectrometry in the positive-ion mode. The electrospray voltage and capillary temperature were 1.6 kV and 250 °C, respectively. The MS scan was acquired within an m/z range of 350 to 2000 in the Orbitrap at a resolution of 70,000. The MS/MS scans were detected in a high-energy collisional dissociation operating mode with 27.0% normalised collision energy. Identical ions that were detected more than two times within 15 s were dynamically excluded.

The collected MS/MS profiles were analysed with Proteome Discoverer software (ThermoFisher Scientific, Waltham, MA, USA) and searched for in the National Center for Biotechnology Information (NCBI) protein database using the following search parameters: fixed modifications, carbamidomethyl (C); variable modifications, oxidation (M); enzyme, trypsin; maximum missed cleavages, 2; peptide mass tolerance, 20 ppm; fragment mass tolerance, 0.6 Da; mass values, monoisotopic.

### 2.8. cDNA Cloning and Red-Change-Related Pigment Protein Gene Sequencing

Total genomic DNA was isolated from the muscle of *P. clarkii*, and DNA quality was assessed by electrophoresis on 1.0% agarose gel. The DNA sequence of PcCRA2 was amplified by degenerate PCR. Based on the highly conserved amino acid sequences MFTT(L/V)(V/I)AA and TAECVYRA among *C. quadricarinatus* crustacyanin A (GenBank: ALC79588.1) and *P. vannamei* crustacyanin A2 (GenBank: XP_027238673.1), two degenerate primers were designated as follows: A2-Fw, 5′-ATGTTTAC(C/A)ACA(C/G)TC(G/A)T(C/T)GCTGCT-3′ and A2-Rev, 5′- TTAAGCTCTGTAGAC(G/A)CA(CT)TC(A/G)GCCG-3′. Degenerate PCR for amplification of A2 used the genomic DNA of *P. clarkii* as the template. The PCR product was purified, ligated to T-Vector pMD19, and sequenced. Total RNA was extracted from the muscle of *P. clarkii* using Trizol column total RNA extraction kit (Sangon Biotech, Shanghai, China). To confirm the introns and obtain the full-length complementary DNA (cDNA) of PcCRA2, reverse transcription PCR was performed using the primers CA2-Fw (5′-ATGTTTACCACTCTAATCGCT-3′) and CA2-Rev (5′- TTAAGCTCTGTAGACGCACTC- 3′).

### 2.9. Circular Dichroism (CD) Spectrophotometry

CD spectra were determined in the range of 190–250 nm by Chirascan circular dichroism spectrophotometry (V100, Applied Photophysics Ltd., Surrey, UK) with a quartz cuvette path length of 0.05 cm. The F1 sample (0.1 mg·mL^−1^) was treated at different temperatures (25, 40, 60, 80, and 100 °C) for 10 min, filtered through a 0.22 µm syringe filter, and scanned three times at 1 nm·s^−1^. The spectral resolution was 0.5 nm, time per point 0.5 s, bandwidth 1.0 nm, and measured temperature 25 °C. The CD intensity is expressed as the mean molar ellipticity (*θ*) (deg·cm^2^·dmol^−1^). The secondary structure data was calculated using CDNN version 2.1 software.

### 2.10. Bioinformatics Analysis

The cloned sequences were first analysed via the NCBI’s online basic local alignment search tool (BLAST). Then, amino acid sequences of PcCRA2 were analysed by ProtParam. Next, the sequences were analysed for identity using DNAman Version 6 (Lynnon Biosoft, San Ramon, CA, USA). The signal peptide was predicted by the Signal program (http://www.cbs.dtu.dk/services/SignalP-4.0/, accessed on 1 September 2021). The self-optimised prediction method with alignment (SOPMA) online software was used to predict secondary structure. Homologous models were generated on the Swiss-model online server. PyMOL was used to analyse the obtained models with the PcCRA2 model from *H. gammarus* chosen as a template (Protein Data Bank code: 1gka). The quality of the model PcCRA2 was assessed by MolProbity and QMEAN server. Finally, the PcCRA2 and astaxanthin were docked using AutoDock Vina 4.0.

### 2.11. Statistical Analysis

All measurements were done in triplicate. The data were analysed by the Statistical Package for the Social Sciences (SPSS) software (version 18.0, SPSS Inc., San Francisco, CA, USA) and are expressed as mean ± standard deviation (SD).

## 3. Results and Discussion

### 3.1. Pigment Protein Extraction and Purification

**Figure 1:**
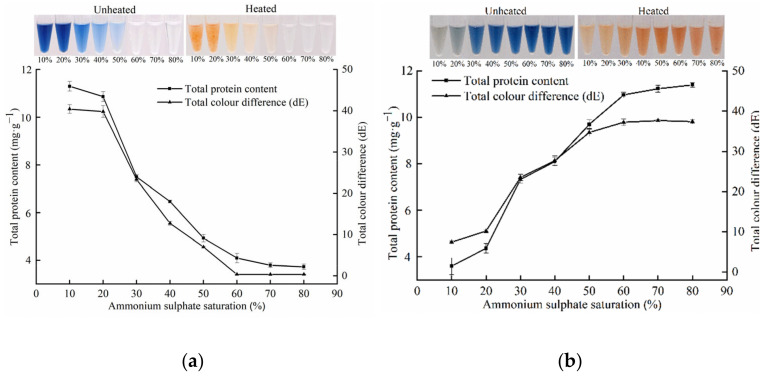
The ammonium sulphate curve of the red-change-related primary pigment proteins: (**a**) the supernatant, (**b**) the precipitate.

Ammonium sulphate precipitation is a classic and widely used method employed to preliminarily purify proteins owing to its low cost, low toxicity to protein, and convenient operation [23,24]. As the saturation of (NH_4_)_2_SO_4_ increases, different undenatured proteins precipitate from crude extracts and can be recovered. Figure 1 shows the ammonium sulphate curve of the red-change-related primary pigment proteins. At (NH_4_)_2_SO_4_ saturation of 10% and 20%, the total protein content of the supernatant decreased, but that of the recovered precipitate increased to 0.36 and 0.44 mg·g^−1^, respectively. After the heat treatment at 100 °C for 10 min, the supernatant turned colour from blue to red; however, there was no significant colour change in the precipitate. These results suggested that partially interfering proteins were removed by 20% (NH_4_)_2_SO_4_ saturation. When the (NH_4_)_2_SO_4_ saturation increased from 20% to 60%, the total protein content and total colour difference (dE) of the supernatant gradually decreased from 1.09 to 0.41 mg·g^−1^ and from 39.82 to 0.37, respectively. In the 60–80% (NH_4_)_2_SO_4_ saturation range, the dE of the supernatant remained almost constant at approximately 0.37, with no noticeable colour transition occurring upon heating at 100 °C for 10 min. This indicated that the remaining protein in the supernatant was not associated with red colour change. Therefore, an (NH_4_)_2_SO_4_ saturation of 20–60% was used to precipitate potential red-change-related primary pigment proteins.

**Figure 2:**
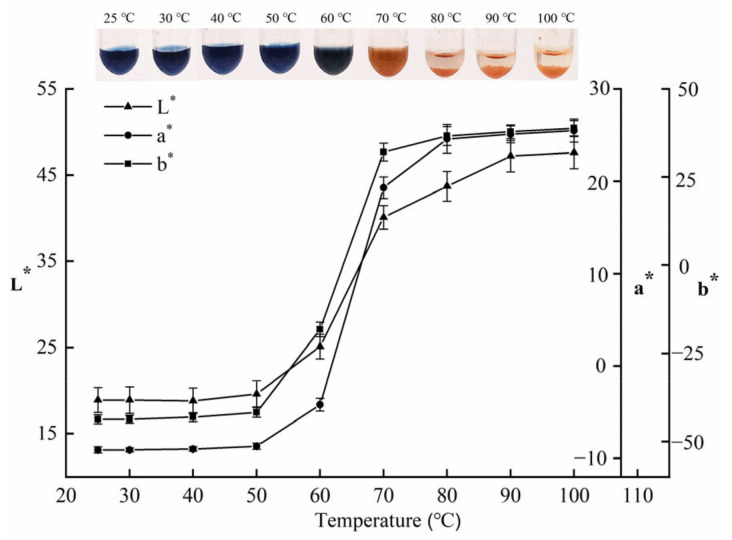
Colour changes of primary pigment proteins were obtained by 20–60% ammonium sulphate precipitation during the heat treatment from 25 to 100 °C for 10 min.

**Figure 3:**
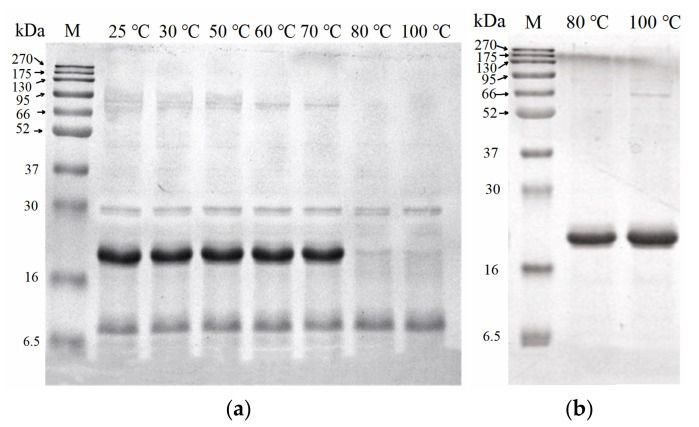
SDS-PAGE analysis of heated primary pigment proteins. (**a**) After heating at 25 to 100 °C for 10 min. (**b**) The precipitate of primary pigment proteins after heating at 80 and 100 °C for 10 min. (Lane M indicates the protein markers.)

To further confirm the red colour change of the primary pigment proteins, the primary pigment proteins were heated at 25 to 100 °C for 10 min (Figure 2). No significant colour change occurred between 25 and 50 °C. At 60 °C, the primary pigment proteins became dark green with an L* value of 25.1, a* value of −4.2, and b* value of −18.2. After thermal treatment at 70 to 100 °C, red colour changes in primary pigment proteins were observed, as well as substantial precipitation from 80 to 100 °C. The colour change of primary pigment proteins increased significantly as the heating temperature increased. This may be related to the denaturation of pigment proteins by high temperatures. In addition, the heating treatment may alter or destroy the interaction between pigment and protein [25]. SDS-PAGE analysis of the supernatants of the above primary pigment proteins from the 25, 30, 50, 60, 70, 80, and 100 °C heat treatments is shown in Figure 3a. A marked protein band with a molecular weight (MW) of 21 kDa was discovered in samples subjected to the heat treatment below 80 °C; however, this disappeared at higher than 80 °C heat treatment. SDS-PAGE analysis of the red precipitate during 80 and 100 °C treatment revealed an evident 21 kDa protein band (Figure 3b). Therefore, 21 kDa protein was presumed to be associated with the red colour change.

**Figure 4:**
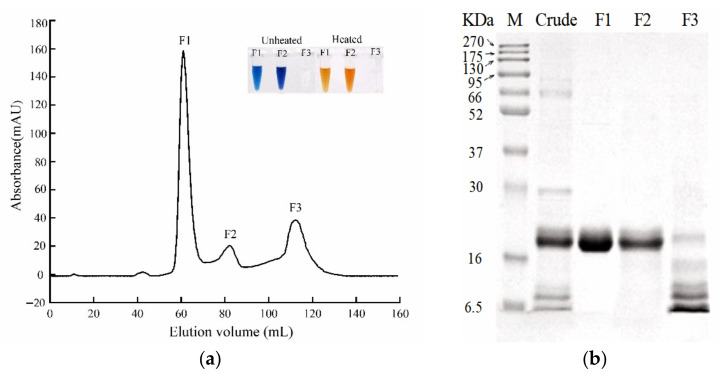
Purification of red-change-related pigment proteins by size exclusion chromatography. (**a**) Elution size exclusion chromatography profile. (**b**) SDS-PAGE profiles of the crude pigment proteins and purified fractions F1, F2, and F3. (Lane M is the protein markers.)

Three fractions (F1, F2, and F3) were isolated from the primary pigment proteins by SEC. The content of F1 was markedly higher than that of F2 and F3 (Figure 4a). After thermal treatment at 100 °C, the red colour change was observed only in F1 and F2; there was no colour change in F3 (Figure 4a). After SDS-PAGE analysis, a single 21 kDa band was observed for F1 and F2, whereas F3 displayed 6.5 to 16 kDa bands but no 21 kDa band (Figure 4b). These results indicated that F1 and F2 were pigment proteins associated with red changes, and both were composed of 21 kDa subunits. The findings were slightly different from those of Garate et al., who reported two major protein bands of 19.2 and 22.4 kDa in a carapace of *P. clarkii* [26]. However, Pan et al. isolated two different red colour-related proteins of 24 and 73 kDa, where 73 kDa protein was a tripolymer composed of three 24 kDa protein units [21]. This possibly occurred because differently coloured crayfish were used in different studies, as various red-change-related pigment proteins of different molecular weights have also been found in other crustaceans. In a European lobster shell, the pigment-protein MWs ranged from 48 to 90 kDa [17]. Two major pigment proteins of 211 kDa and 45 kDa were found in a shell of *P. monodon* [27]. Additionally, Wade et al. found that 21 kDa protein played a key role in colour-related proteins with MWs ranging from 10 to 260 kDa [28]. A 75 kDa red-colour-related protein was purified from the shells of *P*. *vannamei* and *M*. *japonicus* [7,19]. Similar results were obtained for several other crustaceans, including *Carcinus maenas* (38.2 kDa), *Asellus aquaticus L*. (20 kDa), and *Eulimnogammarus cyaneus* (15 and 25 kDa) [29,30,31].

SEC of F1 and F2 revealed a prominent single peak for each, at retention times of 13.02 and 15.95 min, respectively (see Supplementary Materials—Figure S1). The corresponding molecular masses were estimated to be 170 kDa and 43 kDa, respectively, according to the retention time of the protein standard mix. The results of SEC and SDS-PAGE together indicated that F1 and F2 were octamer and dimer proteins composed of 21 kDa subunits, respectively.

### 3.2. Pigment Protein Identification, cDNA Cloning and Sequence Analysis

Peptide sequences of the 21 kDa subunit of F1 and F2 were obtained by Q Exactive LC-MS/MS and compared with data in the NCBI database, respectively. A candidate protein was considered reliable provided the score was greater than 89 (*p* < 0.05). No known proteins with sequences similar to those of the 21 kDa subunits of F1 and F2 from *P. clarkii* were found, but crustacyanins from several shrimp species with scores of greater than 89 were discovered. Among these, *C. quadricarinatus* crustacyanin A was listed in the 21 kDa subunit of F1 and F2 with scores of 821 and 445, respectively (Table 1 and Table S1). The 21 kDa subunit in F1 and F2 was predicted to be homologous and belonged to the crustacyanin family; it was denoted as *P. clarkii* crustacyanin A2 (PcCRA2).

The DNA and cDNA sequences of PcCRA2 were 951 and 573 bp, respectively. The DNA sequence of PcCRA2 contained three exons and two introns, with intron lengths of 112 and 266 bp. The open reading frame of PcCRA2 was 573 bp in length and corresponded to a predicted polypeptide with 190 amino acid residues (21.15 kDa, pI = 5.59). The signal peptide region was present in the amino acid sequence between 1 and 16. The nucleotide sequence data were submitted to the GenBank database under accession numbers MW727506 and MW727507 (see Supplementary Materials—Figure S2).

**Figure 5:**
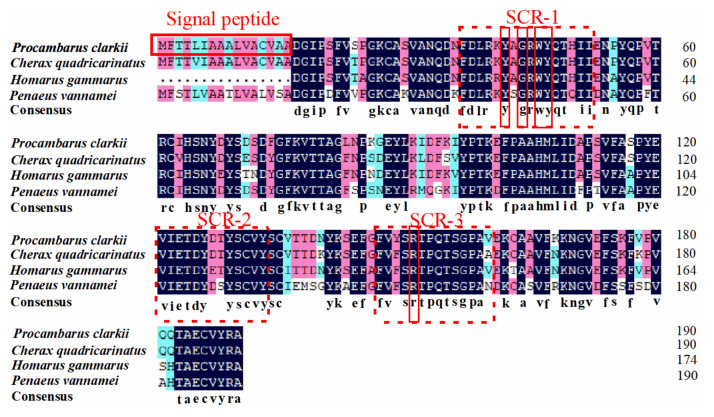
Amino acid sequence multiple alignments of crustacyanin from crustaceans.

A multiple-protein sequence alignment revealed that PcCRA2 was highly homologous to the crustacyanins from other crustacean species (Figure 5). The amino acid sequence of PcCRA2 was 91.58% similar to that of *C. quadricarinatus* crustacyanin A (Accession: ALC79588.1), 87.93% similar to that of *H.* *gammarus* crustacyanin A2 subunit (Accession: P80007.1), and 76.32% similar to that of the *P. vannamei* crustacyanin A2 subunit-like isoform X1 (Accession: XP_027238673.1). The amino acid sequence of PcCRA2 contained three regions that are typical of the lipocalins family, including SCR-1 (G-X-W, X represents any amino acid), SCR-2 (T-D-Y), and SCR-3 (R) [32].

**Figure 6:**
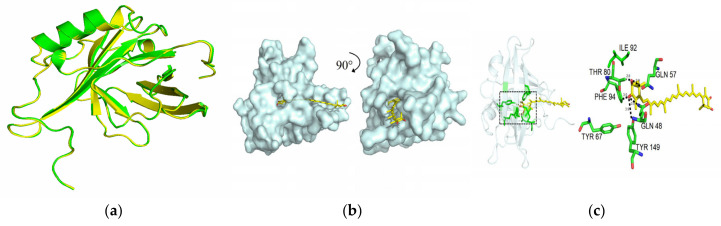
Tertiary structure modelling of PcCRA2. (**a**) Tertiary structure comparison between PcCRA2 (green) and the template *H. gammarus* crustacyanin A2 (yellow). (**b**) Molecular docking diagram of astaxanthin and PcCRA2. (**c**) Key amino acid binding analysis of PcCRA2 and astaxanthin.

The secondary structure prediction of PcCRA2 comprised random coils (42.63%), α-helixes (27.37%), β-sheets (23.68%), and a few β-turn (6.32%), as determined by SOPMA software. On this basis, the tertiary structure of PcCRA2 was modelled on the Swiss-model server using the existing crystal structures of *H. gammarus* crustacyanin A2 subunit as a template (PDA code: 1gka). The Ramachandran plot obtained from MolProbity analysis showed that 93.02% of the residues were located in the most favoured regions, whereas only 0.58% of the residues were in prohibited regions (data not shown). This suggested that the modelled structure of PcCRA2 was sufficiently accurate. Furthermore, the normalised QMEAN4 Z-score for the model PcCRA2 was found to be 0.92, which was in the range of the defined limits of the good quality model. Figure 6a showed that the structural similarity between them was greater than 85%. As expected, PcCRA2 also had the typical topology of the lipocalin family, namely a β-barrel structure composed of eight antiparallel β-strands arranged to form two orthogonal β-sheets [33]. Furthermore, the shape of the barrel-like calyx and the bottom of the barrel were blocked by a conserved tryptophan residue at the N-terminus. The pigment was encapsulated in the protein at one end, with the innermost β-ionone ring close to the bottom and the other end protruding away (Figure 6b). The molecular docking results suggested that seven key amino acid residues—Gln48, Gln57, Tyr67, Thr80, Phe94, Ile92, and Tyr149—were non-covalently combined with the end rings of astaxanthin (Figure 6c). Specifically, hydrophilic residues Gln57 and Thr80 were bonded via hydrogen to the O3 hydroxyl group with hydrogen bonding distances of 2.9 and 2.8 Å, respectively. Two hydrophilic residues, Gln48 and Tyr149, and one hydrophobic residue, Phe94, were close to the O4 carbonyl oxygen and formed three hydrogen bonds. Tyr67 and Ile92 residues were present in the calyx. These hydrogen bonds may have caused a shift in the position of amino acids [34], which was shown by Chayen et al. to generate a conformational change of pigment proteins but without a secondary structural change [5]. However, the end β-ring of astaxanthin underwent a significant twist under hydrogen bonding [35]. Therefore, hydrogen bonding was the predominant molecular force that maintained the stability of the secondary and tertiary structures of PcCRA2.

### 3.3. Pigment Protein Colour Change and Secondary Structure Change

Table 2 shows the colour changes of F1 (1 mg·mL^−1^) after the heat treatment at different temperatures for 10 min. With the temperature increasing from 25 to 100 °C, the lightness (L*), redness (a*), and yellowness (b*) gradually increased from 59.50 to 74.40, −12.57 to 26.03, and −25.67 to 25.70, respectively. The initiation of the colour change of F1 was detected at 40 °C, whereas the visible colour change occurred at 60 °C (*p* < 0.05). The red change was induced upon heating above 60 °C. The trend was consistent with the results of the colour change of the primary pigment proteins during heating.

Britton et al. identified that pigment protein derived from *H. gammarus* became red at 60 °C, but visible red precipitation required a higher temperature [36]. In the present work, the colour change of pigment protein from the *P. clarkii* carapace was closely related to temperature, which was in agreement with the findings of Pan et al., who reported no significant colour change in the pigment protein of *P*. *vannamei* at 30 to 60 °C and a gradual red colour change from 60 °C that was complete above 80 °C [7]. Timme et al. also showed that the pigment-protein colour in the lobster *Jasus lalandii* started to change at 45 °C and turned orange at 65 °C and red at 85 °C [6]. From these results, it appears that similar red-change-related pigment protein colour changes occur upon heating but with different red-change temperatures. This may be due to the unique structures of pigment proteins from different crustacean species [11].

**Figure 7:**
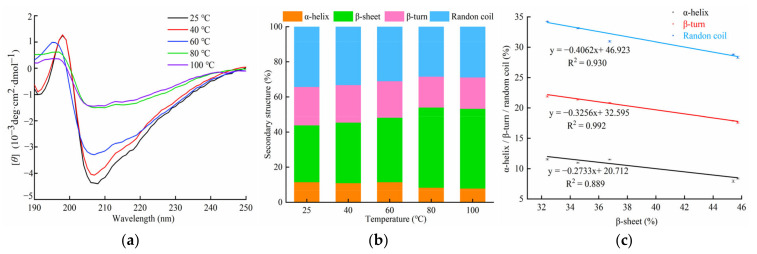
(**a**) CD spectral analysis of F1 heated at different temperatures (25, 40, 60, 80, and 100 °C). (**b**) Changes in the relative content of secondary structural elements of F1 heated at different temperatures (25, 40, 60, 80, and 100 °C). (**c**) Content of α-helices, β-turns, and random coils relative to the β-sheet content of F1 heated at different temperatures (25, 40, 60, 80, and 100 °C).

As can be seen in Figure 7a, the unheated F1 displayed characteristic β-sheet structure peaks, with a positive peak in the region of 195–200 nm and a broad negative peak at 205–215 nm; this was consistent with the results of a previous study by Lin et al. [37]. As the temperature increased, the molar residue ellipticity (*θ*) of the negative peak at 205–215 nm tended to increase (i.e., to smaller negative values), with the effect being more significant at above 60 °C. Additionally, the intensity of the strong positive peak near 195 nm remained unchanged below 60 °C, then gradually decreased with increasing temperature. These changes in the CD spectra indicated that the β-sheet content increased with increasing temperature, suggesting that the structure of the complex gradually unfolded.

Figure 7b shows the relative content of α-helices, β-sheets, β-turns, and random coils of F1 at different temperatures. At 25 °C, the secondary structure of unheated F1 was 11.55% α-helices, 32.40% β-sheets, 21.89% β-turns, and 34.24% random coils, which was different from that of the monomer (21 kDa protein subunit). These changes may have been caused by the interaction of the protein subunits. Compared with the unheated F1, after heating at 40 °C, the content of β-sheets increased slightly, resulting in the initiation of the colour change in F1. At 60 °C, the α-helix content of F1 did not change significantly, whereas the β-sheet content continued to increase. This may have been due to the enhancement of intermolecular hydrogen bonding and the increase in intermolecular protein aggregation, which altered the interaction between the pigment and the protein. A dramatic change in the secondary structural elements occurred at 80 °C, wherein the α-helix content decreased to 8.36% and the β-sheet content increased to 45.72%. However, no significant difference was observed in the relative proportion of secondary structural elements between 80 and 100 °C. Most likely, the disruption of intermolecular hydrogen bonds enhanced the unfolding of the protein structure, resulting in the exposure of hydrophobic amino acid residues from the β-barrel structure [38]. Furthermore, a greater extent of protein-protein aggregation was induced at higher temperatures [39]. The unfolding and aggregation of proteins eventually led to the collapse of the barrel-like calyx and the release of astaxanthin, resulting in a red colour change in the complex [40].

Figure 7c shows the ratio of the content of β-sheets, α-helices, and random coils to the β-turn content for the heat-treated F1. The results suggested that the enhancement in β-sheet content was highly correlated to the decline in α-helix, β-turn, and random coil content (R^2^ = 0.889, R^2^ = 0.992, and R^2^ = 0.930, respectively).

The correlation analysis of the heating temperature, secondary structural elements, and colour parameters are shown in Table 3. This showed that the heating temperature was significantly positively associated with the redness value (a^*^; r = 0.962, *p* < 0.01). Additionally, the heating temperature was positively correlated with the β-sheet content (r = 0.950, *p* < 0.05) and negatively correlated with the content of α-helices, β-turns, and random coils (r = −0.890, r = −0.927 and r = −0.954, respectively; *p* < 0.05).

## 4. Conclusions

In this study, two novel red-change-related pigment proteins of 170 kDa (F1) and 43 kDa (F2) were purified. F1 and F2 were octamer and dimer proteins composed of eight and two 21 kDa subunits, respectively, which all belonged to the crustacyanin family. PCR analysis results showed that the 21 kDa protein subunit was highly similar to *C. quadricarinatus* crustacyanin A and *H.* *gammarus* crustacyanin A2 and was denoted as *P. clarkii* crustacyanin A2. Prediction of the tertiary structure and molecular docking technology revealed several key amino acid residues (Gln48, Gln57, Tyr67, Thr80, Phe94, Ile92, and Tyr149) played an important role in combining with astaxanthin. When heated above 60 °C, F1 exhibited a visible red colour shift, and its β-sheet content increased with increasing temperature while its α-helix content decreased. Furthermore, the correlation analysis showed that the redness value (a*) of F1 was highly correlated with the heating temperature and the β-sheet content. These findings contribute to a better understanding of the mechanism of the red shift of pigment proteins in crustaceans.

## Figures and Tables

**Table 1:** Comparison of the peptide sequences of the 21 kDa subunit of F1 and F2 derived from *P. clarkii* and matched proteins in the NCBI database.

Fraction	Accession	Mass	Score	Description
F1	ALC79588.1	21,541	821	Crustacyanin A (*C. quadricarinatus*)
	ASY04980.1	20,783	218	Crustacyanin A1, partial (*Penaeus longistylus*)
	pdb|1GKA|B	19,942	146	Chain B, The Molecular Basis of the Colouration Mechanism in Lobster Shell. Beta-Crustacyanin at 3.2 A Resolution
	ACL37121.1	7583	134	Crustacyanin C, partial (*C. quadricarinatus*)
	ASY04979.1	20,871	131	Crustacyanin A1, partial (*Penaeus esculentus*)
	ROT83799.1	21,513	118	Crustacyanin subunit A (*P. vannamei*)
F2	ALC79588.1	21,541	445	Crustacyanin A (*C. quadricarinatus*)
	ASY04980.1	20,783	223	Crustacyanin A1, partial (*Penaeus longistylus*)
	pdb|1GKA|B	19,942	209	Chain B, The Molecular Basis of the Colouration Mechanism in Lobster Shell. Beta-Crustacyanin at 3.2 Å Resolution
	ACL37112.1	21,731	196	Crustacyanin-A1 precursor (*Panulirus cygnus*)

**Table 2:** Colour changes of F1 (1 mg·mL^−1^) after the heat treatment at different temperatures (25, 40, 60, 80, or 100 °C) for 10 min.

Temperature (°C)	L*	a*	b*	dE
25	59.50 ± 0.10 d	−12.57 ± 0.12 d	−25.67 ± 0.06 d	
40	59.90 ± 0.20 d	−12.23 ± 0.15 d	−25.37 ± 0.15 d	0.60
60	61.70 ± 0.36 c	1.13 ± 0.15 c	7.17 ± 0.15 c	35.65
80	70.77 ± 0.47 b	23.53 ± 0.35 b	24.13 ± 0.31 b	62.18
100	74.40 ± 0.30 a	26.03 ± 0.21 a	25.70 ± 0.10 a	65.50

Note: Values are represented as mean ± SD (standard deviation), *n* = 3; different lowercase letters (a, b, c, d) indicate significant differences (*p* < 0.05).

**Table 3:** Correlation analysis of heating temperature, secondary structures, and colour parameters of F1.

	Temperature	L*	a*	b*	α-Helices	β-Sheets	β-Turns	Random Coils
Temperature	1	0.950 *	0.962 **	0.951 *	−0.890 *	0.950 *	−0.927 *	−0.954 *
L*		1	0.974 **	0.896 *	−0.976 **	0.969 **	−0.969 **	−0.910 *
a*			1	0.968 **	−0.932 *	0.988 **	−0.982 **	−0.973 **
b*				1	−0.815	0.938 *	−0.917 *	−0.980 **
α-Helices					1	−0.958 *	0.968 **	0.868
β-Sheets						1	−0.997 **	−0.973 **
β-Turns							1	0.957 *
Random coils								1

Note: * indicates significant differences (*p* < 0.05), and ** indicates highly significant differences (*p* < 0.01).

## Data Availability

The datasets generated for this study are available on request to the corresponding author.

## Supplementary Materials

The following are available online at https://www.mdpi.com/article/10.3390/foods11010035/s1, Table S1: Peptide sequences of the 21 kDa subunit of F1 and F2 matched with *Cherax quadricarinatus* crustacyanin A. Figure S1: The molecular weight of native purified pigment proteins F1 and F2 determined by size exclusion chromatography (SEC). Figure S2: Nucleotide sequence and deduced amino acid sequence of PcCRA2.
