# Supplementary material for: Purification and Characterisation of Two Novel Pigment Proteins from the Carapace of Red Swamp Crayfish (Procambarus clarkii)
Source: Foods. 2021 Dec 23;11(1):35. doi: 10.3390/foods11010035 (PMC8750329; doi:10.3390/foods11010035)
Supplement: Supplementary file 1 [file foods-11-00035-s001.zip › foods-1499339-supplementary.pdf]

Table S1. Peptide sequences of the 21 kDa subunit of F1 and F2 matched with *Cherax quadricarinatus* crustacyanin A

Fraction	Start–end	Observed mass (Da)	Calculated mass (Da)	Peptide sequence
F1	28–41	546.5963	1636.7627	CASVANQDNFDLRK
	46–61	682.3455	2044.0166	WYQTHIIENPYQPVTR
	62–77	657.6016	1969.7789	CVHSNYDYSESDYGFK
	78–91	771.3791	1540.7409	VTTAGFNPSDEYLK
	92–100	535.2828	1068.5492	LDFSVYPTK
	101–140	1152.7716	4607.0383	EFPAAHMLIDAPSVFASPYEVIET DYDTYSCVYSCVTTDK
	143–151	538.2638	1074.5135	SEFGFVFSR
	152–169	938.9699	1875.9149	TPQTSGPAAEKCAAVFNK
	163–176	524.2615	1569.761	CAAVFNKNGVEFSK
	177–189	542.6074	1624.8032	FKPVQQTAECVYR
F2	28–41	546.5963	1636.7627	CASVANQDNFDLRK
	46–61	682.3455	2044.0166	WYQTHIIENPYQPVTR
	62–77	657.6016	1969.7789	CVHSNYDYSESDYGFK
	92–100	535.2828	1068.5492	LDFSVYPTK
	143–151	538.2638	1074.5135	SEFGFVFSR

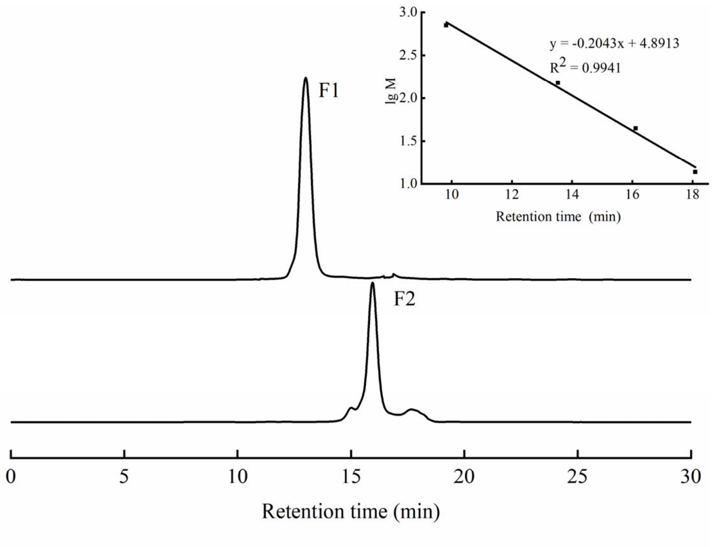

Figure S1. The molecular weight of native purified pigment proteins F1 and F2 determined by size exclusion chromatography (SEC)

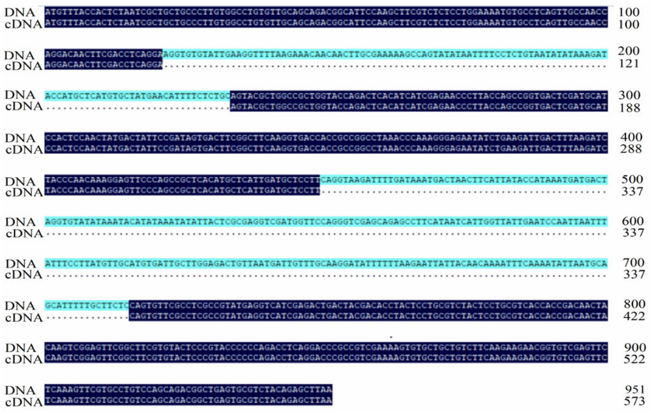

Figure S2. Nucleotide sequence and deduced amino acid sequence of PcCRA2.

DNA of PcCRA2 (951 bp)

ATGTTTACCACTCTAATCGCTGCTGCCCTTGTGGCCTGTGTTGCAGCAGACGGCATTCCAA-
GCTTCGTCTCTCCTGGAAAATGTGCCTCAGTTGCCAACCAGGACAACTTCGACCTCAG-
GAAGGTGTGTATTGAAGGTTTTAAGAAACAACAACTTGCGAAAAAGCCAGTATA-
TAATTTTCCTCTGTAATATATAAAGATACCATGCTCATGTGCTATGAACATTTTCTCTGCAG-
TACGCTGGCCGCTGGTACCAGACTCACATCATCGAGAACCCTTAC-
CAGCCGGTGACTCGATGCATCCACTCCAACTATGACTATTCCGA-
TAGTGACTTCGGCTTCAAGGTGACCACCGCCGGCCTAAACCCAAAGGGAGAATATCTGAA-
GATTGACTTTAAGATCTACCCAACAAAGGAGTTCCCAGCCGCTCACATGCTCATT-
GATGCTCCTTCAGGTAAGATTTTGATAAATGACTAACTTCATTATACCA-
TAAATGATGACTAGGTGTATATAAATACATA-
TAAATATATTACTCGCGAGGTCGATGGTTCCAGGGTCGAGCAGAGCCTTCATAATCATT-
GGTTATTGAATCCAATTAATTTATTTCCTTATGTTGCATGTGATTGCTTGGA-
GACTGTTAATGATTGTTTGCAAGGATATTTTTTAAGAATTATTACAACAAAATTTCAAAA-
TATTAATGCAGCATTTTTGCTTCTCCAGTGTTCGCCTCGCCGTATGAGGTCATCGAGACTGAC-
TACGACACCTACTCCTGCGTCTACTCCTGCGTCACCACCGACAACTACAAGTCGGAG-
TTCGGCTTCGTGTACTCCCGTACCCCCCAGACCTCAG-
GACCCGCCGTCGAAAAGTGTGCTGCTGTCTTCAAGAAGAACGGTGTCGAG-
TTCTCAAAGTTCGTGCCTGTCCAGCAGACGGCTGAGTGCGTCTACAGAGCTTAA

cDNA of PcCRA2 (573 bp)

ATGTTTACCACTCTAATCGCTGCTGCCCTTGTGGCCTGTGTTGCAGCAGACGGCATTCCAA-
GCTTCGTCTCTCCTGGAAAATGTGCCTCAGTTGCCAACCAGGACAACTTCGACCTCAG-
GAAGTACGCTGGCCGCTGGTACCAGACTCACATCATCGAGAACCCTTAC-
CAGCCGGTGACTCGATGCATCCACTCCAACTATGACTATTCCGA-
TAGTGACTTCGGCTTCAAGGTGACCACCGCCGGCCTAAACCCAAAGGGAGAATATCTGAA-
GATTGACTTTAAGATCTACCCAACAAAGGAGTTCCCAGCCGCTCACATGCTCATT-
GATGCTCCTTCAGTGTTCGCCTCGCCGTATGAGGTCATCGAGACTGACTACGACAC-
CTACTCCTGCGTCTACTCCTGCGTCACCACCGACAACTACAAGTCGGAGTTCGGCTTCGTG-
TACTCCCGTACCCCCCAGACCTCAGGACCCGCCGTCGAAAAGTGTGCTGCTGTCTTCAAGAA-
GAACGGTGTCGAGTTCTCAAAGTTCGTGCCTGTCCAGCAGACGGCTGAGTGCGTCTACAGAGCTTAA

Amino acid sequence of PcCRA2 (190 aa)

MFTTLIAAALVACVAADGIPSFVSPGK-
CASVANQDNFDLRKYAGRWYQTHIIENPYQPVTRCIHSNYDYSDSDFGFKVTTAGLNPKGEYLKIDFKIYPTKEFPA
AHMLIDAPSVFASPYEVIETDYDTYSCVYSCVTTDNYKSEFGFVYSRTPQTSGPAVEKCAA-
VFKKNGVEFSKFVPVQQTAECVYRA
